# Parental body mass index and maternal gestational weight gain associations with offspring body composition in young women from the Nutritionists’ Health Study

**DOI:** 10.20945/2359-3997000000516

**Published:** 2022-09-20

**Authors:** Freitas Renata Germano Borges de Oliveira Nascimento, Ana Carolina Junqueira Vasques, Francieli Barreiro Ribeiro, Isabela Solar, Alfredo Shigueo Hanada, Marina Gomes Barbosa, Angélica Marques Martins Valente, Bianca de Almeida Pititto, Ilana Eshriqui, Tito Lívio da Cunha Lopes, Bruno Geloneze, Sandra Roberta Gouvea Ferreira

**Affiliations:** 1 Universidade de São Paulo Escola de Saúde Pública Departamento de Epidemiologia São Paulo SP Brasil Departamento de Epidemiologia, Escola de Saúde Pública, Universidade de São Paulo, São Paulo, SP, Brasil; 2 Universidade Estadual de Campinas Faculdade de Ciências Médicas Laboratório de Investigação em Metabolismo e Diabetes, Gastrocentro Campinas SP Brasil Laboratório de Investigação em Metabolismo e Diabetes, Gastrocentro, Faculdade de Ciências Médicas, Universidade Estadual de Campinas, Campinas, SP, Brasil; 3 Universidade Estadual de Campinas Escola de Ciências Aplicadas Campinas SP Brasil Escola de Ciências Aplicadas, Universidade Estadual de Campinas, Campinas, SP, Brasil; 4 Universidade Federal de São Paulo Departamento de Medicina Preventiva São Paulo SP Brasil Departamento de Medicina Preventiva, Universidade Federal de São Paulo, São Paulo, SP, Brasil; 5 Hospital Israelita Albert Einstein São Paulo SP Brasil Hospital Israelita Albert Einstein, São Paulo, SP, Brasil; 6 Universidade Federal do Piauí Teresina PI Brasil Universidade Federal do Piauí, Teresina, PI, Brasil; 7 Universidade Estadual de Campinas Centro de Pesquisa de Obesidade e Comorbidades Campinas SP Brasil Centro de Pesquisa de Obesidade e Comorbidades, Universidade Estadual de Campinas, Campinas, SP, Brasil

**Keywords:** DOHaD, body composition, obesity, parental BMI, gestational weight gain

## Abstract

**Objective:**

Intrauterine environment can induce fetal metabolic programming that predisposes to adiposity-related chronic diseases in its lifespan. We examined the associations of parental nutritional status and gestational weight gain with offspring body composition in early adulthood.

**Materials and methods:**

This is cross-sectional analysis of female participants of the NutriHS who were submitted to questionnaires, clinical examinations and body composition assessed by DXA. Association of pre-conception parental BMI and maternal gestational weight gain (exposures) with body composition measurements (outcomes) were analyzed using multiple linear models adjusted for Directed Acyclic Graphs-based covariables (maternal and paternal educational level, maternal age, and tobacco, alcohol and/or drugs use). The sample included 124 women (median 28 (24-31) years) with a mean BMI of 25.4 ± 4.7 kg/m^2^.

**Results:**

No association between previous paternal BMI and offspring’s body composition was detected. In the fully adjusted linear regression model, maternal BMI was associated with offspring’s total lean mass (β = 0.66, p = 0.001), appendicular skeletal muscle mass index (ASMI) (β = 0.11, p = 0.003) and fat mass index (FMI) (β = 0.03, p = 0.039). Gestational weight gain was associated with increased offspring’s BMI (OR 1.12 [95% CI 1.02-1.20], p = 0.01). The linear regression model adjusted for maternal age and maternal and paternal education levels showed associations of gestational weight gain with offspring’s ASMI (β = 0.42, p = 0.046), FMI (β = 0.22, p = 0.005) and android-to-gynoid fat ratio (β = 0.09, p = 0.035).

**Conclusion:**

Our findings suggest that preconception maternal BMI could influence lean mass and general adiposity of young adult female offspring and that gestational weight gain could be useful for predicting centrally distributed adiposity.

## INTRODUCTION

High rates of excess body fatness in both sexes are reported worldwide and particularly in women of childbearing age obesity represents a great concern (1,2). Excessive weight gain during pregnancy has been associated with gestational complications, contributing to maternal and infant morbidity and mortality (3). Moreover, during the offspring lifespan, studies have shown that parents’ increased adiposity predicts infant obesity and elevates the cardiometabolic risk later in life (4–6).

Compelling associations of maternal nutritional status and gestational weight gain with offspring adiposity have suggested that these conditions contribute to a vicious cycle of obesity (7–11). Not only genetic factors but epigenetic programming plays an important role for the obesity epidemic (5,6). Our group found that maternal pre-pregnancy body mass index (BMI) was associated with offspring adherence to a processed or to a prudent dietary pattern in adulthood (12). Others reported that lifestyle of the pregnant woman affects her intrauterine environment and may influence the offspring’s risk for adiposity-related outcomes later in life (13). Also, there is evidence that paternal pre-conception exposure to adverse lifestyle factors can affect spermatogenesis by epigenetic mechanisms and that modified gene expressions can be passed onto subsequent generation (5,14). In fact, transgenerational nutrition-linked effects on cardiometabolic risk of the offspring through the male line was previously proposed in cohort studies (15).

Despite the reported associations of parental pre-conception BMI and gestational weight gain with offspring adiposity (4,8–11,16), studies rarely assessed all compartments of the body. Particularly, the increased abdominal fat generates systemic inflammation and insulin resistance which are underlying mechanisms of cardiometabolic diseases (17). Whether a slightly elevated visceral adiposity can deteriorate metabolic profile even in young non-obese adults has been scarcely investigated (18,19). This information could be useful in identifying a subset at mildly increased risk for cardiometabolic diseases and, as a result, intervening earlier to prevent events.

To the best of our knowledge, no study has tested simultaneously associations of parental adiposity and gestational weight gain with body compartments of young adult offspring using dual-energy x-ray absorptiometry (DXA) that provides accurate measurements of body composition (20). We examined whether paternal and maternal pre-conception BMIs and gestational weight gain would be independently associated with DXA-assessed muscle, bone and adipose tissue of the offspring during the early adulthood.

## MATERIALS AND METHODS

### Study protocol and participants

This is a cross-sectional analysis of baseline data of the multicenter Nutritionists’ Health Study (NutriHS), conceived in the School of Public Health, University of Sao Paulo, Brazil, to investigate markers of cardiometabolic diseases. Briefly, the NutriHS has been conducted in selected Brazilian cities, involving undergraduates of Nutrition courses and nutritionists (12). Such particularity of the sample, aware of the relevance of providing good quality data, might be relevant to minimize memory bias. The NutriHS was approved by research ethics committees and informed was consent signed electronically on the specific website (www.fsp.usp.br/nutrihs). The current analysis was based on the data collected in the University of Campinas (Unicamp), São Paulo State, in which the recruitment occurred between 2018 and 2019. Eligibility criteria were female undergraduates or graduates aged 20 to 45 years, with BMI ranging between 18.5 and 39.9 kg/m² and mothers alive. Pregnant and lactating women, those with diabetes, heart, kidney, liver disease, cancer and AIDS or using medications for weight control or affecting glucose metabolism were excluded.

Volunteers responded to structured questionnaires regarding early life events, socioeconomic, lifestyle, dietary data (21,22) and health aspects. Afterwards, they were invited to clinical examination and laboratory procedures. During a visit to the UNICAMP Laboratory of Investigation in Metabolism and Diabetes, participants of the present analysis attended a face-to-face interview, had blood sample collected and body composition assessed. From 127 eligible volunteers (i.e., the offspring), 124 had complete information of all variables of interest for this study.

### Parents’ data and offspring’s early-life and current data

For the present study, we called “early-life data” those conditions occurring pre- and after conception of the participants that could be associated with outcomes in adult life. Considering evidence that parental elevated pre-conception BMIs, as well as excessive gestational weight gain, could trigger epigenetic modifications and influence offspring’s risk, these were variables taken from the questionnaires. Birth weight and duration of breastfeeding were also early-life variables of interest. For answering the questionnaire specially regarding early-life data, participants were oriented to ask for their mothers’ help and to consult birth cards. In most cases, mothers provided the answers but, in case of doubt, the participants could answer “I do not know”. Paternal and maternal ages at conception were obtained in years and their education level was categorized as < 11 or ≥ 11 years. Pre-conception parental BMIs were obtained as continuous variable and further they were categorized as < 25 or ≥ 25 kg/m². Maternal gestational weight gain was obtained as a continuous variable and then was classified in three categories (insufficient, adequate and excessive) based on pre-pregnancy maternal BMI (for BMI < 18.5 kg/m^2^: total weight gain 12.5-18.0 kg; for BMI 18.5-24.9 kg/m^2^: weight gain 11.5-16.0 kg; for BMI 25.0-29.9 kg/m^2^: weight gain 7.0-11.5 kg and for BMI ≥ 30.0 kg/m^2^: weight gain 5.0-9.0 kg) (23).

Additional maternal data were parity (0; ≥ 1 pregnancy), gestational diabetes, hypertension, or other complications (yes; no), tobacco, alcohol and/or drugs use (no; yes) and type of delivery (vaginal; C-section). Offspring’s birth weight and duration of exclusive breastfeeding were collected as continuous variable and categorized into < 2.5; 2.5 and < 3.9; ≥ 4.0 kg and into < 6 months or ≥ 6 months, respectively. Presence of obesity during childhood (no; yes) was based on their mothers’ report.

The offspring variables of interest for this study were age (years), skin color (white; non-white); tobacco, alcohol, drugs’ use (no; yes), education level (undergraduate; graduate), family income (< 6; ≥ 6 minimum wages; one minimum wage was approximately BR$ 725 or US$ 181) and leisure physical activity practice (no; yes). Physical activity was evaluated using the short version of the International Physical Activity Questionnaire (24) validated for Brazilians (25).

### Clinical and biochemical data

Systolic and diastolic blood pressure levels were taken after resting seated, in triplicate using a mercury sphygmomanometer. After overnight fasting, blood samples were collected for several determinations. Glucose was measured by the glucose oxidase method. Total cholesterol, HDL-c and triglycerides were determined by enzymatic colorimetric methods and LDL-c was calculated using the Friedewald equation.

### Anthropometric measurements and body composition

The offspring’s weight was measured using a digital scale (Filizola^®^, São Paulo, Brazil) with 0.1 kg of precision, and height by a fixed stadiometer with 0.1 cm precision. BMI was calculated and nutritional status classified according to the WHO (WHO, 2015) (26). An inelastic tape was used to obtain calf circumference at the largest protuberance of the calf and waist circumference at the midpoint between the last rib and the iliac crest.

Body composition of the NutriHS participants was assessed using DXA (GE Lunar iDXA^®^ with EnCore software, Madison, WI, USA) that provided measurements of adipose, muscle and bone compartments. Total lean mass, total fat mass, trunk fat, android and gynoid fat (used to calculate android-to-gynoid fat ratio – A/G) and visceral adipose tissue (VAT) mass (in grams) were obtained. Availability of arms and legs masses allowed calculating the appendicular skeletal muscle mass (ASM). Fat mass index (FMI) and ASM index (ASMI) were calculated dividing total fat mass and ASM per squared height, respectively, and were expressed in kg/m^2^. According to the manufacturer’s instructions, regions of interest were determined manually; the densitometer was routinely calibrated.

### Statistical analyses

To detect a difference equal to 50% common standard deviation, with a test power of 80% and a significance level of 5%, the number of participants per group calculated was 62, resulting in a sample size of 124. The Kolmogorov-Smirnov test was used to verify normality of continuous variables, which were described as means (standard deviations – SD) and medians (q25-q75 ranges – IQR). For variables with normal distribution, Student t test was used to compare variables of the offspring stratified according to their parental BMI categories (< or ≥ 25 kgm^2^), ANOVA with Bonferroni’s *post hoc* test to compare these variables according to categories of gestational weight gain (insufficient, adequate and excessive) and Pearson’s coefficient to test correlations. Non-parametric tests (Mann-Whitney and Kruskal-Wallis tests and Spearman’s coefficient) were used for non-normal distributed data. Categorical variables were described as frequencies and compared using chi-squared or Fisher’s exact test. Odds ratios and 95% confidence intervals were estimated.

**Supplementary Figure 1:**
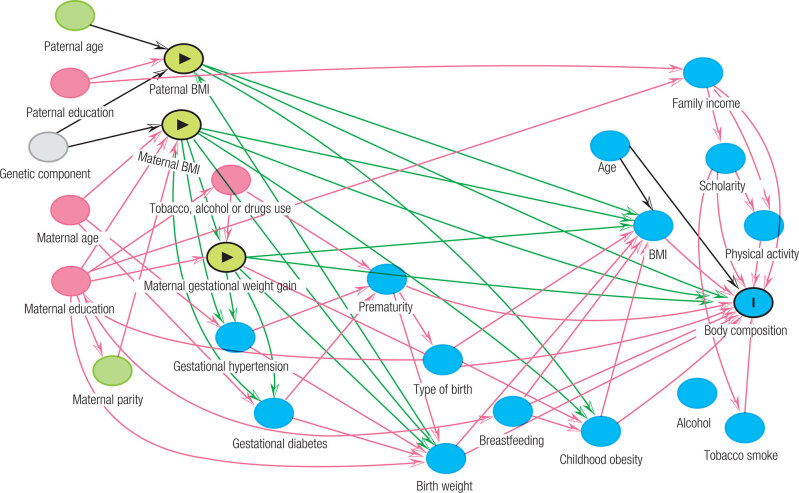
Directed Acyclic Graph (DAG) for the association between previous parental body mass indexes and gestational weight gain with body composition. Minimal sufficient adjustment set included maternal education, maternal age, paternal education and maternal use of tobacco, alcohol and/or drugs. BMI: body mass index; (>) exposure; (I) outcome.

Associations between dependent variables (body composition parameters and BMI) and independent variables (early-life data) were analyzed using multiple linear regression. Early-life data of main interest were parental preconception BMIs and gestational weight gain (exposures) while offspring’s body composition measurements (continuous variables) were the outcomes. Directed Acyclic Graph (DAG) was employed to select covariables for adjustments in multiple regression models (27). DAG is causal diagram based on scientific assumptions about the relationship between variables using mathematically rigorous methodology for reducing bias and avoiding overadjustment (28). The DAG was built using the Daggity software (dagitty.net) and it is available in Supplementary Figure 1. The set of minimal sufficient adjustments based on the DAG was maternal and paternal education, maternal age, and tobacco, alcohol and/or drugs use.

Maternal gestational weight gain, VAT, android fat FMI and bone mineral density were log-transformed before linear regression analyses. Crude associations of parental BMIs and gestational weight gain with total fat, gynoid fat, android fat, A/G fat ratio, FMI, ASMI and VAT were initially tested. Model 1 was adjusted for maternal and paternal education levels and maternal age; in model 2, tobacco, alcohol and/or drugs use were added. Statistical significance was set at the p-level of 5%. Statistical Package for the Social Sciences^®^, version 24.0 was used for analyses.

## RESULTS

The sample of 124 NutriHS participants included 52% of nutritionists and 48% of undergraduates. Main characteristics of the study participants are shown in Table 1. Most participants reported white skin color, were single and practiced regular physical activity. Ninety-four percent were breastfed and 22% had exclusive breastfeeding for 6 months. Thirty-one percent reported having been overweight in childhood or adolescence. Five participants referred having been followed a vegetarian diet for at least 6 months. Most of their parents attended elementary or middle school. Reported frequencies of overweight or obese prior to conception was higher among the fathers (39.0%) than among the mothers (10.5%). Mothers with less education were more likely to have daughters with overweight or obesity than those with higher education [OR 2.29, 95% CI (1.03-5.10)].

**Table 1:** Main sociodemographic characteristics of the NutriHS sample (n = 124) and parental data

		Median (IQR)	Range
**Offspring data**			
	Age (years)	28 (24-31)	19 – 42
	Body mass index (kg/m^2^)	25 (21-28)	18.5 – 39.9
Nutritional status (%)			
	Eutrophic	50.0	
	Overweight	34.0	
	Obese	16.0	
Skin color (%)			
	White	72.0	
	Non-white	28.0	
Physical activity (%)			
	Regular practice	71.0	
	No	29.0	
Marital status (%)			
	Single	72.0	
	Married or stable union	28.0	
Living condition (%)			
	Live with their parents	46.0	
	Live with their partners	34.0	
	Live alone	20.0	
Family income (%)			
	1 to 5 minimum wages	46.0	
	≥6 minimum wages	54.0	
Family history (%)			
	Cardiovascular diseases	68.0	
	Dyslipidemia	60.0	
	Hypertension	50.0	
	Diabetes	38.0	
**Parental data**			
Parental preconception BMI ≥ 25 kg/m^2^ (%)			
	Father	39.0	
	Mother	10.5	
Paternal education (%)			
	Elementary or middle school	62.7	
	Higher education level	37.3	
Maternal education (%)			
	Elementary or middle school	65.2	
	Higher education level	34.8	

Maternal BMI before pregnancy was not correlated to gestational weight (r = 0.11, p = 0.26). Tobacco, alcohol and/or drugs’ use during pregnancy was reported by 13.2% and 90% had no clinical complications. Offspring’s mean birth weight was 3.2 ± 0.5 kg. There was a positive correlation between maternal prepregnancy BMI and offspring birth weight (r = 0.27; p < 0.01) and present BMIs (r = 0.26; p < 0.01).

Clinical, biochemical and body composition data of the participants, stratified according to their parental BMI categories and to gestational weight gain, are shown in Table 2. Gestational weight gain was insufficient in 36%, adequate in 34% and excessive in 30% mothers. On average, all subgroups showed normal values of blood pressure, anthropometric and metabolic variables and most variables did not differ between subgroups. Mean values of BMI (29.0 ± 5.3 *versus* 24.8 ± 4.4 kg/m^2^, p < 0.01) and calf circumference (41.2 ± 4.0 *versus* 36.7 ± 3.2 cm, p < 0.01) of daughters from mothers with increased preconception BMI were higher than the counterparts. Offspring from mothers who gained excessive weight during pregnancy had higher mean plasma glucose than the others (83.7 ± 4.8 *versus* 80.3 ± 5.9 and 82.5 ± 5.5 mg/dL, respectively for excessive, insufficient, and adequate, p < 0.05). Regarding body composition, offspring from mothers with increased BMI before pregnancy had higher mean values of total lean mass (45.4 ± 4.4 *versus* 37.7 ± 4.7 kg, p < 0.01, respectively) ASM (20.9 ± 2.4 *versus* 16.5 ± 2.5 kg, p < 0.01, respectively) and ASMI (7.5 ± 0.8 *versus* 6.4 ± 0.8 kg/m^2^, p < 0.01, respectively).

**Table 2:** Clinical, biochemical and DXA-assessed body composition data of 124 participants according to preconception parental body mass indexes and maternal gestational weight gain

	Paternal BMI (kg/m^2^)		Maternal BMI (kg/m^2^)		Gestational weight gain		
	<25	≥25	<25	≥25	Insufficient	Adequate	Excessive
**Clinical and biochemical data**							
Systolic BP^a^ (mmHg)	110 (100-110)	110 (100-110)	110 (100-110)	110 (100-120)	110 (100-110)	110 (100-110)	110 (100-120)
Diastolic BP^a^ (mmHg)	70 (70-80)	76 (70-80)	70 (70-80)	80 (70-80)	70 (70-80)	70 (70-80)	80 (70-80)
Body mass index (kg/m²)	25.1 ± 5.1	24.9 ± 3.4	24.8 ± 4.4	**29.0 ± 5.3** **	24.1 ± 4.3	25.5 ± 4.8	26.5 ± 4.7
Waist circumference^a^ (cm)	81.0 (74.0-93.0)	81.8 (74.0-87.1)	80.1 (74.0-92.0)	87.5 (83.2-100)	76.8 (72.0-89.2)	83.0 (75.5-92.8)	83.0 (76.0-93.0)
Calf circumference (cm)	37.3 ± 4.0	37.2 ± 3.1	36.7 ± 3.2	**41.2 ± 4.0** **	36.6 ± 3.2	37.5 ± 3.7	37.7 ± 3.5
Fasting plasma glucose	81.9 ± 5.6	81.5 ± 5.6	82.0 ± 5.6	80.3 ± 4.9	80.3 ± 5.9	82.5 ± 5.5	**83.7 ± 4.8** *
HOMA-IR^a^	1.2 (0.8-1.7)	1.0 (0.7-1.4)	1.2 (0.8-1.7)	1.0 (0.9-1.3)	1.2 (0.7-1.7)	1.2 (0.8-1.7)	1.0 (0.7-1.4)
Total cholesterol^a^ (mg/dL)	173 (160-194)	160 (144-181)	167 (151-192)	161 (134-165)	165 (147-194)	163 (148-177)	165 (148-193)
LDL-cholesterol^a^ (mg/dL)	95 (77-111)	87 (76-108)	92 (76-111)	92 (76-99)	87 (76-111)	90 (76-98)	96 (78-111)
HDL-cholesterol^a^ (mg/dL)	60 (50-69)	56 (49-64)	60 (50-68)	52 (39-56)	57 (49-68)	56 (49-63)	61 (49-67)
Triglycerides^a^ (mg/dL)	81 (61-119)	73 (60-81)	76 (60-103)	65 (53-86)	77 (59-100)	76 (59-111)	66 (60-86)
**Body composition data**							
Total lean mass (kg)	38.3 ± 5.0	38.8 ± 5.3	37.7 ± 4.7	**45.4 ± 4.4** **	37.3 ± 3.7	39.1 ± 5.5	39.5 ± 5.5
ASM (kg)	16.7 ± 2.7	17.1 ± 2.9	16.5 ± 2.5	**20.9 ± 2.4** **	16.0 ±1.9	17.4 ± 2.9	17.6 ± 3.0
ASMI (kg/m^2^)	6.4 ± 0.9	6.5 ± 0.9	6.4 ± 0.8	**7.5 ± 0.8** **	6.2 ± 0.7	6.7 ± 0.9	6.7 ± 0.9
Total fat mass (%)	38.1 ± 7.1	37.3 ± 6.6	38.0 ± 7.1	40.6 ± 8.3	36.4 ± 7.4	37.9 ± 7.1	40.1 ± 6.8
Fat mass index (kg/m^2^)	7.8 (6.8-11.9)	8.6 (6.6-11.5)	8.4 (6.7-11.3)	11.4 (7.2-13.2)	7.2 (6.010.6)	8.5 (6.9-11.5)	10.1 (8.1-11.6)
Android fat^a^ (%)	34.7 (27.4-46.1)	36.3 (25.0-45.8)	35.4 (27.3-46.1)	42.8 (31.1-54.9)	33.3 (24.2-45.9)	35.9 (28.9-44.6)	38.5 (31-48.1)
Gynoid fat (%)	43.2 ± 6.9	43.0 ± 5.9	43.4 ± 7.0	45.6 ± 6.4	41.5 ± 7.1	43.4 ± 6.7	45.3 ± 6.7
Android-to-gynoid fat ratio	0.8 ± 0.2	0.8 ± 0.2	0.8 ± 0.2	0.9 ± 0.2	0.8 ± 0.2	0.8 ± 0.2	0.9 ± 0.1
Trunk fat^a^ (%)	35.4 (29.6-45.2)	37.2 (27.7-44.1)	36.65 (29.1-45.2)	42.7 (30.7-53.1)	34.3 (26.2-44.0)	36.7 (30.1-45.7)	38.6 (33.9-46.6)
Visceral adipose tissue^a^ (g)	134 (82-465)	178 (87-432)	156 (88-463)	332 (130-734)	118 (80-435)	243 (105-493)	224 (94-561)
Total lean mass (kg)	38.3 ± 5.0	38.8 ± 5.3	37.7 ± 4.7	**45.4 ± 4.4** **	37.3 ± 3.7	39.1 ± 5.5	39.5 ± 5.5
Bone mineral density (g/cm^2^)	1.1 (1.1-1.2)	1.1 (1.1-1.1)	1.1 (1.1-1.2)	1.2 (1.2-1.3)**	1.1 (1.1-1.1)	1.1 (1.1-1.2)	1.1 (1.1-1.2)

Data expressed as mean ± standard deviation or median and q25-q75 ranges in parenthesis. Variables compared using Student t test or Mann-Whitneyᵃ and ANOVA or Kruskal Wallis.ᵃ

* p < 0.05 *versus* insufficient maternal gestational weight gain.

** p < 0.01 *versus* Maternal BMI < 25 kg/m^2^.

BMI: body mass index; BP: blood pressure; HOMA-IR: Homeostasis Model Assessment – Insulin Resistance; ASM: appendicular skeletal muscle mass; ASMI: appendicular skeletal muscle mass index.

**Supplementary Figure 2:**
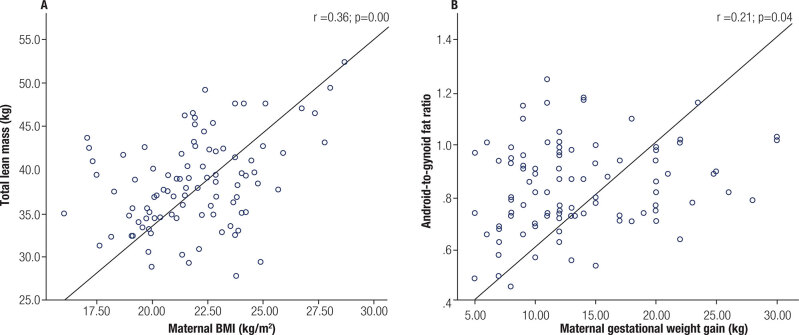
Scatter plots showing correlations of measurements of body composition of the NutriHS participants and preconception maternal body mass index (^A^) and gestational weight gain (^B^)

Selected correlations between early-life exposures and current body composition parameters of the offspring were shown in Supplementary Figure 2. Preconception paternal BMI was not correlated to the outcomes, but preconception maternal BMI showed to be directly correlated to total lean mass (r = 0.36; p < 0.001) and ASMI (r = 0.28; p < 0.001). Gestational weight gain was correlated to offspring’s percent of total fat (r = 0.22; p = 0.03) and trunk fat (r = 0.24; p = 0.02), FMI (r = 0.27; p = 0.01) and A/G fat ratio (r = 0.21; p = 0.04), but not to VAT (r = 0.12, p = 0.26). No correlation between parental BMIs (father: r = 0.03, p = 0.75; mother: r = 0.12, p = 0.24) and gestational weight gain (r = 0.19, p = 0.07) with bone mineral density was detected.

In linear regression models, preconception maternal BMI were associated with offspring total lean mass (β = 0.66, p = 0.001), ASMI (β = 0.11, p = 0.003) and FMI (β = 0.03, p = 0.039) independently of maternal and paternal educational level, maternal age, and tobacco alcohol, drugs’ use (Table 3, model 2). Gestational weight gain showed to be associated with waist circumference after the same adjustments (β = 0.07, p = 0.033, not shown in tables). Gestational weight gain was associated with several measures of the adipose tissue: total fat (β = 3.51, p = 0.046), android (β = 0.19, p = 0.019) and A/G fat ratio (β = 0.09, p = 0.039) and with FMI (β = 0.22, p = 0.006).

**Table 3:** Linear regression models for the associations of offspring’s body composition measurements with pre-conception maternal body mass index and gestational weight gain

			Crude			Model 1			Model 2		
		Exposures	β	95% CI	p-value	β	95% CI	p-value	β	95% CI	p-value
		**Maternal BMI**									
Muscle tissue											
	Total lean mass (kg)		0.72	(0.33;1.11)	0.000	0.65	(0.26;1.05)	0.001	0.66	(0.26;1.05)	0.001
	ASMI (kg/m^2^)		0.11	(0.03;0.17)	0.004	0.11	(0.04;0.18)	0.004	0.11	(0.04;0.18)	0.003
Adipose tissue											
	Total fat (%)		0.10	(-0.48;0.68)	0.739	0.31	(-0.27;0.88)	0.293	0.31	(-0.26;0.88)	0.280
	Android fat (%)#		0.13	(-0.01;0.04)	0.338	0.22	(-0.00;0.48)	0.101	0.02	(-0.00;0.48)	0.092
	Gynoid fat (%)		0.03	(-0.53;0.59)	0.923	0.22	(-0.34;0.78)	0.442	0.22	(-0.33;0.78)	0.430
	Android-to-gynoid fat ratio		0.01	(-0.00;0.21)	0.233	0.01	(-0.00;0.02)	0.091	0.01	(-0.00;0.20)	0.084
	Visceral adipose tissue (g)#		0.04	(-0.05;0.13)	0.369	0.07	(-0.02;0.16)	0.135	0.07	(-0.02;0.16)	0.125
	Fat mass index (kg/m^2)^#		0.02	(-0.01;0.05)	0.158	0.03	(0.00;0.06)	0.046	0.03	(0.00;0.06)	0.039
		**Gestational weight gain**									
Muscle tissue											
	Total lean mass (kg)		1.19	(-1.33;3.71)	0.350	1.49	(-0.98;3.97)	0.233	1.45	(-1.03;3.94)	0.248
	ASMI (kg/m^2^)		0.39	(-0.03;0.80)	0.068	0.42	(0.01;0.83)	0.046	0.41	(0.00;0.82)	0.050
Adipose tissue											
	Total fat (%)		3.30	(-0.30;6.90)	0.072	3.60	(0.14;7.05)	0.042	3.51	(0.06;6.96)	0.046
	Android fat (%)#		0.18	(0.17;0.34)	0.031	0.19	(0.04;0.35)	0.017	0.19	(0.03;0.34)	0.019
	Gynoid fat (%)		3.07	(-0.42;6.57)	0.084	3.41	(0.04;6.78)	0.047	3.33	(-0.04;6.70)	0.053
	Android-to-gynoid fat ratio		0.08	(0.00;0.16)	0.046	0.09	(0.01;0.17)	0.035	0.09	(0.00;0.17)	0.039
	Visceral adipose tissue (g)#		0.29	(-0.30;0.87)	0.331	0.34	(-0.23;0.91)	0.236	0.32	(-0.24;0.89)	0.256
	Fat mass index (kg/m^2^)#		0.20	(0.04;0.36)	0.014	0.22	(0.07;0.38)	0.005	0.22	(0.06;0.37)	0.006

Model 1: adjusted for maternal and paternal education levels (<11; ≥ 11 years) and maternal age (years).

Model 2: model 1 plus maternal tobacco, alcohol and/or drugs use (yes; no).

# Variables were log-transformed.

CI: confidence; ASMI: appendicular skeletal muscle mass index.

In a model adjusted for parental education and maternal age (model 1), an association of gestational weight gain with ASMI was detected (β = 0.42, p = 0.046). No association between parental BMIs and gestational weight gain with VAT or bone mineral density was detected.

## DISCUSSION

This is the first study to investigate simultaneously associations of parents’ adiposity prior to conception and maternal gestational weight gain with DXA-determined body compartments of a young adult offspring. Both maternal preconception BMI and gestational weight gain were independently associated with the nutritional status of a female offspring. While maternal preconception BMI was associated with measures of muscle mass and general adiposity, only maternal gestational weight gain was associated with parameters of central adiposity. The latter could reinforce the importance of adequate weight gain during pregnancy in order to diminish a risky deposition of fatness in adulthood. This is of great clinical relevance given the dramatic trend of obesity nowadays.

Our findings are supported by the Developmental Origins of Health and Disease theory in which adaptive responses occur in the period of plasticity (the thousand-day life period starting at conception). Diverse exposures – including parental nutrition – were shown to induce epigenetic alterations favoring certain phenotypes throughout life (29,30). Our study is in line with others suggesting that even the preconception nutritional status of the mother could influence the adult offspring body composition after adjustment for several potential confounders. The DAG-based approach was opportune since a possible effect of the gestational weight gain on the offspring’s body composition could be considered in our causal inference path.

Positive associations between fat and lean mass with maternal preconception BMI were previously reported in children (8,9,10,31,32) but also an inverse association was described (11,33). In adult offspring, a study used skinfolds to estimate body fat (16) and in another, body composition was assessed by DXA, but age group was younger (19 to 21 years) (11) than ours (20 to 45 years) and we considered several potential confounders. We expected to find association of maternal preconception BMI and central adiposity measures as our group had observed using data obtained in another center of the NutriHS (34). Such lack of association has been attributed to the smaller sample analyzed in the present study. On the other hand, our findings of direct associations of maternal BMI with offspring’s ASMI and FMI are relevant since these indexes provided information about body composition corrected by the individual height. Their use is particularly important for mixed populations with high variation in anthropometric characteristics like the Brazilian example. We speculate that the associations found could be attributed in part to heredity, which requires investigation in studies with appropriate design.

On the other hand, no association between preconception paternal BMI and offspring body composition was detected. Previous evidence indicated that obese fathers could exhibit sperm epigenetic alterations that, once transferred to the oocyte at fertilization, could favor increased adiposity and cardiometabolic risk in the offspring (10,14,35,36). However, gender-based differences in obesity inheritance have been described (37). Inconsistencies could be also attributed to different samples characteristics and approaches to assess body composition. In addition, pre-conception paternal data may have a higher degree of measurement error compared with maternal ones. Therefore, we confirmed the need for further investigations in this regard.

As far as gestational weight gain is concerned, its association with offspring body adiposity was previously reported when anthropometric measurements were used (4,16,38). Despite widely used to estimate body adiposity, anthropometry did not provide accurate measurements of fat distribution. Even so, a significant association with waist circumference, adjusted for several confounders, was detected in our study. A novel aspect added by us was that, in a female adult offspring, gestational weight gain was associated with DXA-assessed measurements of fat compartment. The most significant association found was with centrally distributed fatness which is of greater interest in identifying cardiometabolic risk. Adjustments included in linear regression models were relevant since the impact of socioeconomic status and tobacco use during pregnancy for weight gain and offspring nutritional status in childhood has been demonstrated (39). Dietary data during pregnancy were unavailable in our study. A maternal diet rich in fat and sugar could trigger epigenetic changes in the offspring that would favor a pro-inflammatory condition as well as preference for hyper-palatable low-nutritious foods (40) contributing to excessive weight gain throughout life. It would be expected that daughters born from mothers with excessive gestational weight gain would have a worse metabolic profile which was not supported in our study. The lack of differences between NutriHS participants stratified by their mothers’ adequacy of weight gain could be attributed to characteristics of our sample, composed of relatively young individuals who were aware of the relevance of a healthy lifestyle.

To explain how gestational weight gain could be associated with body adiposity of adults, it was proposed that increase of maternal fat deposits and hyperinsulinemia could alter the intrauterine environment in order to develop fetal adipocytes. Increased transfer of free fatty acids and other substrates (4,21,41) to fetus should contribute to adiposity by increasing fetal capacity for the formation of new adipocytes (4,42,43). Changes in maternal composition of gut microbiota may be an underlying mechanism; it was described that obese pregnant women and those with excessive gestational weight gain exhibit changes in microbiota composition as well as their offspring (44).

We also found a direct association of gestational weight gain with the ASMI. A higher weight gain should be dependent of a higher energy intake; this condition would promote an intrauterine environment prone the development of insulin target tissues, such as the skeletal muscle. This hypothesis require investigation in studies with appropriate design.

Our study has several limitations. Our sample size was limited by inclusion criteria. Given the relatively small sample size, current data could be viewed as exploratory. The use of DAG was essential to include the minimal sufficient adjustments in regression analyses for avoiding bias and overadjustment. The selected sample, consisting of low-risk, well educated women with nutrition literacy, impedes generalizing our results to samples with different characteristics. Such homogeneity and the healthy status should have contributed to not disclosing differences between subgroups. A strength was the assessment of body compartments using DXA in early adulthood far from menopause. Considering its ability to identify central fat accumulation, which is strongly associated with cardiometabolic diseases, we have suggested this technique as a strategy to identify at-risk women before occurrence of metabolic abnormalities. Other limitations were the cross-sectional approach that is not appropriate to establish a causal relationship between the exposure and long-term outcomes and the use of questionnaires to reconstruct clinical history of the participants. Memory bias regarding the early life data is also a limitation of or study and others involving retrospective data. However, to minimize errors, only participants whose mothers were alive were included since it was shown that women are able to inform about pre-gestational weight with acceptable precision almost 30 years later (45).

In conclusion, our findings suggest that preconception maternal BMI could influence lean mass and general adiposity of young adult female offspring and that gestational weight gain could be also useful to predict of centrally distributed adiposity. We reinforce the relevance of nutritional care of women before conception and during pregnancy. Avoiding elevated BMI and excessive gestational weight gain could contribute to minimizing an at-risk body composition for cardiometabolic diseases.
